# Death of Dr. Joshua R. Lothrop—Proceedings of the Erie County Medical Society, July 22

**Published:** 1869-07

**Authors:** M. G. Potter

**Affiliations:** Secretary


### Death of Dr. Joshua R. Lothrop—Proceedings of the Erie County Medical Society, July 22.

Members present—Drs. Miner, White, Boardman, Mackay, Chace, Diehl, Ayer, Hopkins, Shaw, Ring, Little, Willoughby, Barnes, Cronyn, Mixer, Bartlett, Green, Gay, Samo, Nichell, Loomis, Brown, Jansen, Wetmore, Strong, Smith, Taylor, O'Brian, Rogers, Storck, Tobie and Potter.

Upon taking the chair, Dr. Miner spoke as follows:

Gentlemen:—It is my painful duty to announce the death of our much respected and worthy friend and professional brother, Dr. Joshua Rich Lothrop. He died this morning, at the residence of a relative, in Plymouth, Mass., where he had gone with his wife, to obtain the repose of country life, and to enjoy the care and society of near friends, hoping thus to regain, in some degree, at least, his former health and vigor; but, alas! he has fallen suddenly from our ranks, and is dead.

My feelings of personal grief and sorrow are too deep for words, and I must leave for you the expression of sadness which the members of the profession all share in the death of one so endeared to them, and so worthy their regard. Dr. Lothrop was a native of Massachusetts, where he spent most of his life. He was a graduate of Dartmouth College, in the year 1844, and in medicine a graduate of the Medical Department of Harvard University in 1852. Early in his professional life he received appointment to the Rainsford Island Hospital, and served with distinguished ability in that institution for a number of years. He came to our city in 1858, and commenced private practice, rapidly gaining the esteem and confidence of the profession, and endearing himself as a physician in many of the most appreciative and intelligent families of the city. As you all know, he has been connected with our public institutions during his entire life among us, and in all the duties of the most responsible positions, has shown himself wise and faithful. During the three years' absence in Europe of Prof. Charles A. Lee, Dr. Lothrop occupied the chair of *Materia Medica* in the Buffalo Medical College, and proved himself a teacher of rare scholarship, of extensive and varied knowledge. As surgeon to the Buffalo General Hospital he won for himself much professional reputation, and has left a record of faithfulness and worth, worthy the highest ambition. He was President of this

Society in 1867, and has received many tokens of respect and favor from the profession, who have always delighted to do him honor. As a private practitioner he enjoyed the confidence of the profession in great degree, and was often called in difficult, dangerous and obscure cases, as one eminently worthy of confidence and trust.

He was greatly admired and beloved by the families who received his professional attentions, and those who knew him best, loved him most; they will mourn his loss as irreparable. Dr. Lothrop was a man of great mind; an original, independent thinker, a true philosopher. He had canvassed the whole field of human knowledge with great care, and rested his beliefs upon the firmest rocks. He said, on my taking leave of him: "I am not anxious for the future; I only fear a long invalidism." He had a true, generous, unselfish nature, and his pure life and character require no eulogy.

He was the idol of a home circle—of a devoted wife, and of a large number of relatives and friends; but I dare not encroach upon the sanctity of their sorrow. You are invited to take such action as you deem proper upon such occasion.

DR. J. P. WHITE arose to bear testimony to the exceeding appropriateness of the remarks just made and to express profound sorrow at the loss of a devoted Professional Brother. The early intimacy which existed between DR. LOTHROP and himself was recalled with pleasure and only tended to impress the many excellencies of the deceased more firmly on his memory. We have lost in him a courteous gentleman and professional scholar. I therefore desire to offer the following:

*Whereas*, It has pleased God to remove by death our friend and fellow member, DR. JOSHUA R. LOTHROP, whom we honored for his professional skill, and loved for his gentleness, modesty and unvarying kindness, and who, in a comparatively brief period, had established in this city a high professional character, and won the friendship of all his brother practitioners, and whose memory is especially endeared to the younger members of the profession as a judicious counsellor and faithful friend,

*Resolved*, That this Society has heard of his sudden death with sincere regret.

*Resolved*, That this Society looks back with great satisfaction on his honorable and successful career as an able and accomplished practitioner and teacher of medicine and surgery, and are filled with emotions of sorrow that a life so promising should have been thus suddenly cut short.

*Resolved*, That we, his fellows, tender our sympathy to the family and relatives of the deceased in the painful bereavement which they have sustained.

*Resolved*, That a properly attested copy of these resolutions be sent to the family of the deceased and that a copy also be furnished to the *Medical and Surgical Journal* and daily papers of this city for publication.

DR. GAY said that DR. LOTHROP was a man whom we all respected and loved. He had lived long enough among us to gain the esteem of his professional associates and also to establish himself in the confidence and esteem of a large number of our best families. He had heard almost every member of this Society express admiration of him during his life time. His career was brief but successful. He heartily approved of the resolutions which had been offered by Dr. White, and moved their adoption.

DR. BOARDMAN concurred with the spirit and tenor of the resolutions and bore testimony to the uniform courtesy and kindness which marked the professional and social career of DR. LOTHROP.

DR. STRONG arose to second the motion of Dr. Gay. Death is said to love a shining mark. It seems that the archer, in this instance, has shown regard for the mark and ability to hit it. It is comparatively rare that we are called to mourn the loss of such a medical man as DR. LOTHROP. He was remarkable in his modesty and in his accomplishments. We can hardly call to mind one of his age who possessed a mind of such judicial fairness. This characteristic must have made its impression on the minds of all his professional associates. His dispassionate estimate of medical questions rendered his judgment always valuable and his frequent discussions in this and the city Society interesting to all who heard them.

Dr. Cronyn arose with no little sorrow to express his assent to the resolutions that had been offered. He had enjoyed the extreme pleasure of frequent conversations with Dr. Lothrop and they had confirmed the esteem in which the deceased was so universally held. Upon leaving the city Dr. Hamilton paid a handsome tribute to the memory of Dr. Lothrop. He said: "Court his acquaintance, Doctor, you will find him a gentleman and a medical scholar of the first order."

Dr. Ring said that Dr. Lothrop's example was a most noble one. His regard for the young practitioner was worthy of him and must cause him long to be remembered by them. To men of his own age his regard for medical ethics was worthy of imitation. His fame was a just fame. He was a safe adviser in consultation and an excellent practitioner.

Dr. Jansen said that he was more familiar with Dr. Lothrop than with any other member of the profession. He had learned to admire him, and when he heard of his death he felt not only that the profession of Buffalo had lost one of its bright lights, but that he had himself met with a great personal bereavement.

Dr. Storck said that in behalf of the younger practitioners of the city he was anxious to bear testimony to the appropriateness of the resolutions and to acknowledge that in Dr. Lothrop we have lost a true and valuable friend, a competent adviser, and one who was always ready to assist whenever an opportunity offered. Dr. Lothrop was especially endeared to the younger members of the profession. In him we have lost an excellent counsellor and an unselfish friend.

The resolutions were adopted and the meeting adjourned.

M. G. Potter, Secretary.

---

The venerable Charles D. Meigs, M. D., one of the most distinguished medical men of the day, died yesterday at his residence in Delaware county. Dr. Meigs was a native of Georgia, but since 1820 he has resided in this city. In the year 1840 he accepted a Professorship in Jefferson Medical College, and for twenty years he labored with much success in that institution. Dr. Meigs was connected with the obstetrical department of the Pennsylvania Hospital for about ten years, and he was well known, both in the Old and New World as a clear and forcible writer on medical subjects, and his published works are considered standard authorities in the medical profession. He leaves a large circle of warm personal and professional friends to mourn his death, and the medical profession loses one of its most distinguished ornaments.—*Philadelphia Telegraph, June 23.—Medical Gazette.*

